# Interferon and autoantigens: intersection in autoimmunity

**DOI:** 10.3389/fmed.2023.1165225

**Published:** 2023-05-09

**Authors:** Brendan Antiochos, Livia Casciola-Rosen

**Affiliations:** Division of Rheumatology, Johns Hopkins University, Baltimore, MD, United States

**Keywords:** autoantibody, autoimmunity, interferon, innate immunity, autoantigen

## Abstract

Interferon (IFN) is a key component of the innate immune response. For reasons that remain incompletely understood, the IFN system is upregulated in several rheumatic diseases, particularly those that feature autoantibody production, such as SLE, Sjögren’s syndrome, myositis and systemic sclerosis. Interestingly, many of the autoantigens targeted in these diseases are components of the IFN system, representing IFN-stimulated genes (ISGs), pattern recognition receptors (PRRs), and modulators of the IFN response. In this review, we describe features of these IFN-linked proteins that may underlie their status as autoantigens. Note is also made of anti-IFN autoantibodies that have been described in immunodeficiency states.

## Introduction

Autoantibodies arise in a wide array of immune-mediated diseases, including both organ-limited and systemic forms of autoimmunity (1, 2). Some autoantigens are organ-specific molecules that are expressed preferentially or even uniquely in the affected organ [e.g., thyroid-specific proteins in autoimmune thyroid disease (3)]. In contrast, antigens targeted in systemic autoimmune rheumatic diseases are frequently ubiquitously expressed, and perform a variety of essential cellular functions (2). The antigens most commonly targeted by antibodies in systemic rheumatic diseases are nuclear antigens, including proteins, nucleic acids, and nucleoprotein complexes (2). The mechanisms responsible for targeting these broadly distributed autoantigens are incompletely characterized, but are likely numerous and overlapping. Here, we will review the relationship between autoantibodies and the IFN system, highlighting the enrichment of IFN-linked antigens in systemic autoimmune rheumatic diseases, and potential explanations for their targeting by autoantibodies.

**Figure 1:**
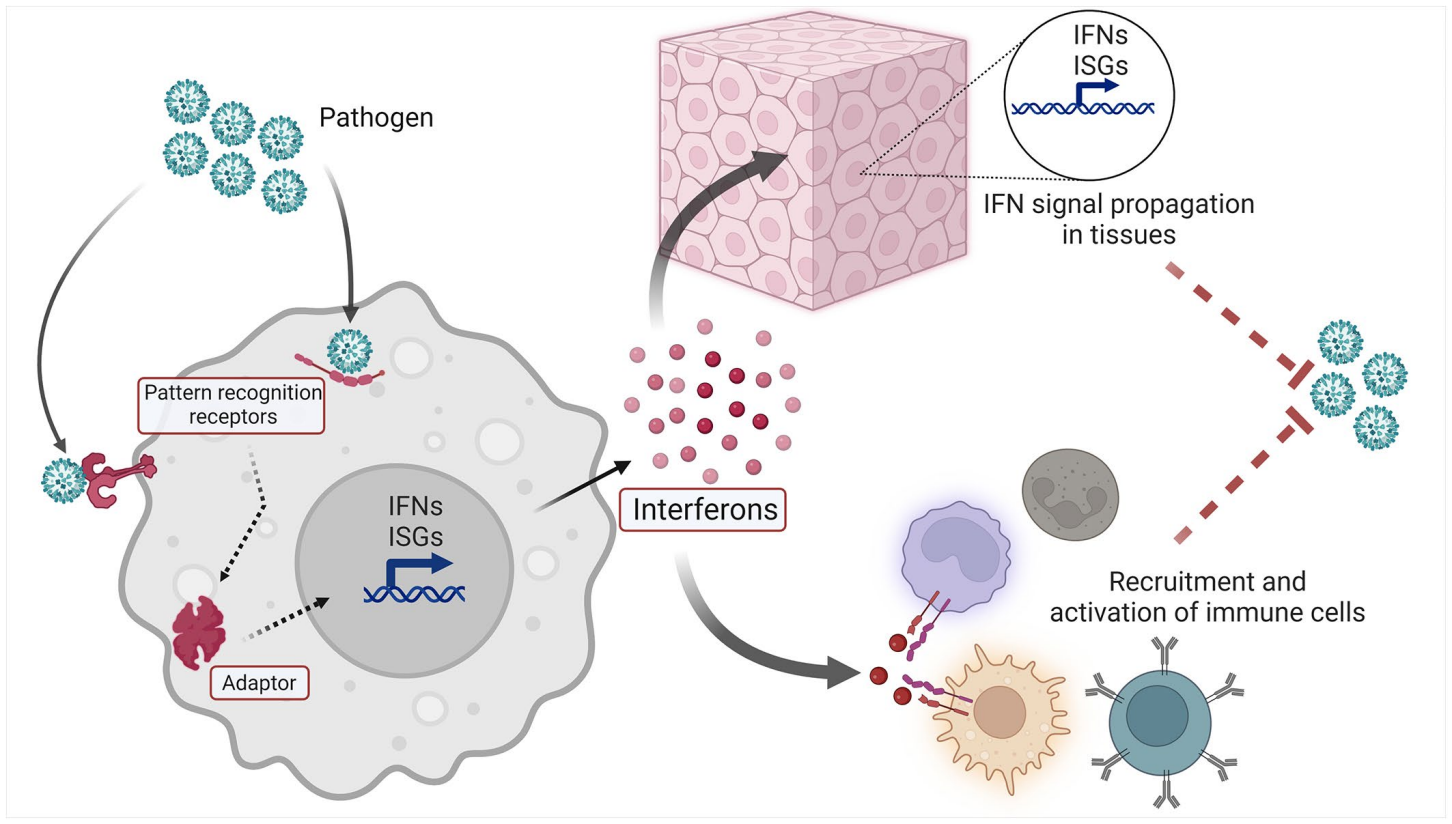
Schematic overview of the IFN system.

The IFN system (Figure 1) is a molecular network that perform host defense functions. Three types of IFNs are found in humans: type I IFNs are expressed by and act on nearly every cell type, type II IFN is more specific for immune cells, and type III IFNs mainly act on epithelial and endothelial cells at mucosal surfaces (4–6). Cell-intrinsic IFN signaling constitutes a primordial layer of innate immunity, enabling resident tissue cells to recognize and respond to a variety of microbial pathogens and nonmicrobial threats. Thus IFN induction within activated cells leads to IFN signaling in neighboring cells via IFNs and second messengers, and in both cases ISG induction occurs. IFNs also perform important cell-extrinsic functions, and are able to shape the immune response by influencing the behavior of immune cells (5).

The upstream elements of the IFN system are innate pattern recognition receptors (PRRs), which recognize an array of Damage and Pathogen Associated Molecular Patterns (DAMPs and PAMPs, respectively) (7). Innate sensors are found in various compartments of the cell, including the endosome (e.g., TLR7), cell surface (e.g., TLR4), and cytoplasm [e.g., cyclic GMP-AMP synthase (cGAS) (8)]. The interaction of ligand and sensor leads to subsequent activation of downstream signaling adaptors, which include molecules such as stimulator of interferon genes (STING), mitochondrial antiviral-signaling protein (MAVS) and MyD88 (8). Activated adaptors then promote signaling through various kinases and transcription factors, which ultimately trigger the expression of Interferon Stimulated Genes (ISGs) and IFNs themselves. This expression of IFNs and ISGs results in both autocrine and paracrine cellular effects. Secreted IFNs bind their cognate receptors on the cell surface, leading to intracellular signaling via the JAK/STAT pathway and expression of ISGs and IFNs in IFN-activated cells (9). In this manner, the IFN system propagates a danger signal rapidly throughout an affected tissue or organ, readying resident parenchymal cells for host defense functions, and influencing the cellular immune response that follows.

Dysregulation of the IFN system has long been recognized as a feature of many autoimmune rheumatic diseases, most notably systemic lupus erythematosus (SLE), Sjögren’s syndrome (pSS), dermatomyositis (DM), and systemic sclerosis (SSc) (10–12). Upregulation of IFNs and ISGs has been observed both in the circulation and the target organs of patients with these diseases. In SLE, IFN I upregulation has been associated with increased markers of serologic and clinical disease activity. Interestingly, longitudinal studies have demonstrated that IFN expression is relatively stable despite changes in disease activity over time (13–17). In pSS, IFN expression has been linked to higher prevalence of autoantibodies and hypergammaglobulinemia, and increased lymphocytic infiltration of salivary tissues (18). Upregulation of IFN has been observed in many types of inflammatory myopathy, with IFN I upregulation particularly notable in DM muscle biopsies (19). Dysregulation of type I, II and III IFNs have all been observed, although the relative degree to which a specific IFN type is activated compared to others varies among individuals (19–23). In addition to the idiopathic rheumatic diseases, dysregulation of IFN has been identified as the driver of genetically-derived interferonopathy syndromes such as Aicardi–Goutières syndrome (AGS) and STING-associated vasculopathy with onset in infancy (SAVI); it is noteworthy that these genetic syndromes present with clinical features that often overlap with those of idiopathic rheumatic diseases (24). Taken together, these observations suggest that IFNs play a key role in the pathogenesis of the autoimmune rheumatic diseases. Consistent with this, therapeutic targeting of IFN has already shown promise in some patients, and is an area of ongoing research (25).

## IFN-induced expression of autoantigens

An ISG is any gene whose expression is increased in response to IFN signaling; there are hundreds of such genes in human cells (26). Many autoantigens are included among the ISGs, suggesting that IFN-responsiveness may be involved in the development of this autoantibody subset. Notable among the IFN-induced autoantigens is Ro52 (encoded by the TRIM21 gene), which is targeted by autoantibodies in many rheumatic diseases, including pSS, SLE, DM, SSc and overlap syndromes (27). An important pathologic function of these antibodies has been defined - maternal anti-Ro52 antibodies demonstrate pathogenic function by mediating congenital heart block (28). In SLE, antibodies against Ro52 are associated with higher levels of circulating IFN I (29, 30). Ro52 is an IFN-induced E3 ligase that targets various substrates for removal via proteasomal degradation. In response to viral infection, Ro52 downregulates the innate immune response by enhancing clearance of the key transcription factor IRF3 (31, 32). Ro52 also promotes antiviral function by serving as a sensor of cytoplasmic IgG, marking intracellular viral-IgG immune complexes for proteasomal clearance (33, 34). Ro52 is therefore both a key regulator of the innate immune response and a functional component of the IFN pathway. It provides an important example of an antigen against which tolerance may be lost due to dysregulated IFN signaling. In this scenario, upregulated antigen expression in the setting of inflammation likely promotes the frequency with which the induced protein is displayed by antigen presenting cells, thereby increasing the likelihood that autoreactivity might occur. Continued expression of IFN in the affected organs would ensure sustained elevated levels of ISG antigens, fueling the propagation phase of such an autoimmune response. It is noteworthy that ISG upregulation caused by interferogenic stimuli (e.g., viral infection) facilitates additional intermolecular interactions that may also lead to breaking of tolerance against Ro52 or other relevant ISGs.

## DNA binding molecules

Several PRRs are included among the autoantigens targeted in rheumatic diseases. While some of these PRR antigens are also IFN-inducible, others are not. Among these non-IFN-induced antigens is Ku - a heterodimeric complex composed of Ku70 and Ku80 subunits that is targeted by autoantibodies in several autoimmune rheumatic diseases (35, 36). In SLE, anti-Ku antibodies have been reported at prevalence of 9.8–20.5% (35, 37). Anti-chromatin antibodies have been identified at greater prevalence among SLE patients with anti-Ku antibodies: anti-chromatin antibodies were found in 72.7% of anti-Ku positive versus 43.9% of anti-Ku negative patient sera in one study (*p* < 0.0001) (37). Anti-Ku antibodies have also been found in association with autoantibodies against additional DNA repair proteins, including DNA-PK, Mre11, WRN and PARP (35). While clearly implicated in DNA repair responses, the Ku complex has also been shown to serve as a cytoplasmic DNA sensor, translocating from the nucleus to the cytoplasm and binding dsDNA of various sorts (38, 39). Recently, Tao et al. demonstrated that cytoplasmic Ku interacts with cGAS to promote condensate formation and IFN signaling in response to cytoplasmic dsDNA (40). These findings raise the intriguing possibility that intermolecular interactions occurring in the context of DNA repair in the nucleus might underlie the targeting of Ku and related autoantigens in SLE, and that Ku may interact with other potential autoantigens in the cytoplasm.

A similar scenario has been observed in the case of poly(ADP-ribose) polymerase (PARP), an additional component of the cellular DNA damage response that is targeted as an autoantigen in SLE and other autoimmune conditions (41, 42). PARP1 translocates to the cytoplasm upon viral infection, where it PARylates cGAS. Interestingly, in contrast to the pro-IFN effect of Ku, this PARylation was reported to inhibit cGAS signaling (43). In addition, the catalytic subunit of DNA-dependent protein kinase (DNA-PKcs), has also been identified as a sensor of cytoplasmic DNA (44), and recently demonstrated to negatively regulate cGAS via phosphorylation (45). It is noteworthy that DNA-PKcs, PARP and Ku are all translocated to the cytoplasm in the setting of dsDNA sensing, and together modulate the cell-intrinsic IFN I response generated by cGAS. Activation of the cytoplasmic dsDNA sensing pathway may therefore represent a stimulus that triggers antigenic changes in these proteins that are relevant to SLE pathogenesis.

RNA polymerase III (POL III) is a well-described autoantigen targeted in 15.3–26.6% of systemic sclerosis patients (46, 47). This enzyme transcribes a variety of noncoding RNA molecules required for routine cellular functions (48). However, its role in activating the IFN system is much less appreciated. A specific function for POL III in the innate immune response was reported by Chiu et al., who showed that POL III converts cytosolic dsDNA into 5′-ppp RNA, which is subsequently detected by RIG-like receptors (RLRs), generating a MAVS-dependent IFN response (49). Thus, Ku, PARP and POL III are all involved in the innate response to cytoplasmic dsDNA. The altered subcellular localization and interactions that occur in the setting of cytoplasmic dsDNA sensing therefore might represent additional mechanisms that could contribute to loss of tolerance against these antigens in autoimmune diseases characterized by an aberrant IFN I response.

## Oligomerizing innate sensors

Several additional autoantigens combine the characteristics of IFN-induced expression, nucleic acid binding, and a third feature specific to their activation: oligomerization. Recent findings from this interesting autoantibody group are reviewed below.

Antibodies against a 140 kDa protein were first described in a Japanese cohort of patients with clinically amyopathic DM (50). The identity of this autoantigen was later demonstrated to be melanoma differentiation-associated protein 5 (MDA5) (51). The initial clinical phenotype described in association with MDA5 antibodies was that of mild muscle involvement, with severe pulmonary manifestations and a variety of cutaneous findings; additional cohort studies have yielded a broader spectrum of clinical manifestations (52). MDA5 is a member of the RIG-Like Receptor (RLR) group of cytoplasmic dsRNA sensors that promote antiviral IFN I production. Upon sensing long dsRNA, MDA5 assembles into filamentous oligomers that activate MAVS and trigger downstream IFN I signaling (53–55). Like Ro52, MDA5 expression is induced by IFN and interestingly, these two antibodies are often targeted together in this subset of DM patients. As MDA5 is both an IFN-inducible and an interferogenic protein, its dysregulation could readily contribute to sustained IFN signaling. Indeed, gain of function mutations in the gene encoding MDA5 (IFIH1) have been identified in patients with interferonopathy syndromes as well as SLE (56–58). Strong IFN I upregulation has been identified in anti-MDA5-associated DM (59), and some have proposed labeling this syndrome an acquired type I interferonopathy (60).

IFI16 is an IFN-inducible dsDNA sensor in the AIM-like receptor (ALR) family (61). Similar to Ku, IFI16 translocates from the nucleus to the cytoplasm upon dsDNA sensing, where it promotes IFN signaling through STING (61). Similar to MDA5, IFI16 also assembles into filamentous oligomers when activated by dsDNA (62). Anti-IFI16 antibodies have been identified in SLE and pSS patients, and are associated with more severe disease features (63–66).

Absent in melanoma 2 (AIM2), another IFN-inducible dsDNA sensor in the ALR family, activates apoptosis-associated speck-like protein containing a caspase-recruitment domain (ASC) upon dsDNA sensing, triggering inflammasome assembly and IL-1/18 secretion (67). We recently identified anti-AIM2 autoantibodies in SLE. These frequently co-occurred with anti-IFI16 and anti-dsDNA antibodies, as well as disease activity markers (68). Autoantibodies targeting ASC (which is also IFN-inducible) have been identified in patients with inflammatory diseases, and anti-ASC antibodies demonstrated a pathogenic ability to enhance inflammasome activation in recipient phagocytes in mice (69).

A noteworthy feature common to the 3 autoantigens MDA5, IFI16 and AIM2 is that they are all IFN-inducible innate sensors of double stranded nucleic acids. Their activation leads to the generation of large, filamentous oligomers of protein and bound nucleic acid ligand. Sustained activation of these sensors at a disease site is one potential explanation for their targeting by autoantibodies. Indeed, our own observation of activated filamentous IFI16 present in the salivary tissues of some pSS patients supports this concept (70). In addition, cytoplasmic interaction of AIM2 and dsDNA has been detected in cell lines derived from pSS salivary tissue (71), and we observed both IFI16 and AIM2 bound to neutrophil extracellular trap DNA in SLE renal tissues (68). These findings provide compelling additional evidence that DNA-bound sensors are present at sites of disease activity.

The presence of oligomerized sensors coupled to nucleic acid ligands may lead to the generation of novel epitopes not found in the monomeric forms, or may increase the potential for autoreactivity via the increased valence present in oligomers that are conveyed to immune cells at sites of immune activation. These autoantigens may therefore represent key molecules whose activation causes pathogenic inflammatory signaling in affected organs, as well themselves being targets of the autoimmune response. Future studies are warranted to examine whether these and/or other autoantibodies serve as biomarkers that identify subsets of patients in whom such innate signaling pathways are especially relevant to disease initiation or propagation. Insights from such studies will likely inform the more effective use of IFN-specific therapies.

## Interferons as autoantigens

In addition to the spectrum of intracellular autoantigens associated with systemic autoimmune rheumatic diseases, antibodies against extracellular antigens have also been described in a variety of scenarios. Anti-cytokine antibodies have been observed in patients with SLE and other rheumatic diseases, and also in viral infection and immunodeficiency states (72). In SLE, antibodies against type I, II and III IFNs have been observed (73). These authors found that antibodies against type I IFNs had a neutralizing function, and patients with blocking anti-type I IFN antibodies demonstrated normalized IFN expression levels. Conversely, SLE patients with anti-IFN II antibodies suffered from more severe disease manifestations, including upregulation of type I IFNs. Antibodies against IFNs were also measured in patients with pSS at a comparable prevalence, but were not observed as often in RA.

Nearly 20 years ago, neutralizing antibodies against type II IFN were recognized in patients suffering unusual, severe mycobacterial infections (74, 75). Since that time, several hundred cases of anti-IFN-gamma-autoantibodies (AIGA) have been reported in patients presenting with a variety of infections. In addition to mycobacterial disease, salmonella, varicella, and fungal species have also been recorded, making AIGA an antibody-mediated form of acquired immunodeficiency. Recent studies in SARS-CoV2 have strengthened the evidence that anti-IFN autoantibodies have functional consequences in the setting of infection, as antibodies directed against type I IFNs have been measured in patients who suffer severe disease outcomes from COVID19 (76–78). These observations suggest that, in the setting of infection, anti-IFN antibodies constitute a potentially treatable form of immunodeficiency that renders the host more susceptible to infection. Conversely, in the setting of autoimmune diseases such as SLE, it remains less clear whether anti-IFN antibodies contribute to disease pathogenesis, or serve as markers of aberrant IFN signaling.

## Conclusion

Autoantibodies target a multitude of cellular antigens, and diverse mechanisms are likely responsible for their targeting through the humoral immune response. Several autoimmune rheumatic diseases feature upregulation of IFN signaling along with autoantibodies directed against components of the IFN system. These IFN-linked autoantigens include ISGs, DNA-binding proteins, and oligomerizing pattern recognition receptors. Pathogenic activation of these IFN system components may underlie their status as autoantigens, and these autoantibodies might therefore indicate patients in whom the antibody-targeted antigens play critical roles in driving IFN activation. Antibodies against IFNs themselves mediate increased susceptibility to some infections and represent a form of acquired immunodeficiency mediated by humoral autoimmunity.

## Author contributions

BA and LC-R contributed to conceptualization and writing of the manuscript. All authors contributed to the article and approved the submitted version.

## Funding

BA is supported by NIH K08AR077100.

## Conflict of interest

The authors declare that the research was conducted in the absence of any commercial or financial relationships that could be construed as a potential conflict of interest.

## Publisher’s note

All claims expressed in this article are solely those of the authors and do not necessarily represent those of their affiliated organizations, or those of the publisher, the editors and the reviewers. Any product that may be evaluated in this article, or claim that may be made by its manufacturer, is not guaranteed or endorsed by the publisher.
